# To Add B, or Not to Add B: That Is the Question

**DOI:** 10.3390/ph19101588

**Published:** 2026-10-08

**Authors:** Marvin A. Soriano-Ursúa, Andrei Biţă

**Affiliations:** 1 Academia de Fisiología y Sección de Estudios de Posgrado e Investigación, Escuela Superior de Medicina del Instituto Politécnico Nacional, Plan de San Luis y Diaz Mirón s/n., Alc. Miguel Hidalgo, Mexico City 11340, Mexico; 2 Faculty of Pharmacy, University of Medicine and Pharmacy of Craiova, 2 Petru Rareş Street, 200349 Craiova, Romania; andrei.bita@umfcv.ro

Incorporating boron (B) into bioactive molecules is not a question in nature. Moreover, some boron-containing compounds (BCCs) have been suggested as inducers of the origin of important biomolecules such as RNA [1,2,3]. In fact, there are several BCCs with proven bioactivity and potential biomedical applications. Examples include boric acid, which regulates growth in some fungi and plants; boromycin, which acts as an antibiotic; and natural fructoborate (found in some seeds and fruits), regulating the metabolism of mammals, including humans [4,5]. Moreover, some dietary BCCs are clearly involved in human health, and their derivatives have medical applications ranging from the prevention of some diseases to theragnostic agents [Contributions 1 and 2].

However, more than two centuries after its discovery as an element, only in recent years B has been included in innovative chemical reactions, and the number of synthetic BCC has increased notably. Boronic acids and boron-containing clusters could be considered among the most promising scaffolds in this century for designing new bioactive agents to address the global burden on human pathologies [6]. Despite the high interest in developing BCC as drugs, the full characterization of these compounds is only now emerging, following FDA approval for five of them as drugs and the availability of pharmacovigilance data for their use in humans, as well as the identification of some other BCCs tested in mammals (including humans) [Contribution 3].

Hence, this Special Issue, titled “Boron-Containing Compounds: Identification, Synthesis, Biological Actions and Pharmacology”, is a global collection (with authors from Australia, Canada, India, Italy, Japan, Mexico, Moldova, Poland, Romania, Turkey, the United Kingdom, the United States of America and Uruguay) of recent advances in the field. It reflects growing interest not only in the physiological role of BCCs, but also in their study as potential drugs, from the molecular details and properties of new BCCs which could confer them advantages over boron-free compounds to their potential and tested activity against human diseases, and their mechanisms of action.

Some of these advances come from theoretical studies [7,8]. Toshev et al. provided comparative thermodynamic insight into the deprotonation behavior of BCCs as proteasome inhibitors under physiological conditions, suggesting that the two hydroxyl groups are not equivalent during deprotonation, providing a physicochemical framework for the design of next-generation BCCs with potential in cancer therapy [Contribution 4]; while Velayutham et al. carried out computational analyses suggesting that tavaborole could be repurposed to act on nitrosative stress and the nitric oxide balance in venous leg ulcers [Contribution 5].

Interest in new medical applications of well-known natural BCCs also continues to grow, supported by current findings [9,10]. One example is the study of boric acid supplementation in cyclophosphamide-induced damaged uterus and fallopian tubes in rats, suggesting a potential protective role in those organs [Contribution 6]. Similarly, sodium pentaborate pentahydrate exerts selective anticancer effects on colorectal cancer cells through the induction of apoptosis, cell cycle arrest, and suppression of key oncogenic pathways [Contribution 7].

Regarding the synthetic BCCs, other approaches have extended the therapeutic applications of BCCs beyond cancer, infections or atopic dermatitis, as is the case for the FDA-approved drugs [6]. Thus, BCCs are crossing barriers: for example, some sugar-BCCs with structural similarity to topiramate are active in the mouse brain as potential antiseizure agents, presumably acting through more than one mechanism of action, including ionic flux regulation [Contribution 8]. In a separate study, synthetic fructoborate showed a potential role in the structural remodeling of isoproterenol-induced cardiac hypertrophy, probably also related to ionic flux. Further studies are required to establish its long-term safety, pharmacokinetics, and potential therapeutic relevance [Contribution 9].

Other borylated monosaccharides are active against cancer; beyond that, the real therapeutic potential of these borylated drugs lies in their switch-on/switch-off activation under boron neutron capture therapy (BNCT) radiotherapeutic conditions [Contribution 10].

Other BCCs are being tested beyond the initial applications. Although B-clusters have been attractive as BNCT agents [11,12], Dávila et al. tested several carborane-erlotinib analogs and found that lipophilicity and electronic features correlated with cytotoxic and inhibition of Epidermal Growth Factor Receptor activity, providing structure–activity relationships against glioblastoma [Contribution 11]. BODIPYs, known for labeling [13], are evolving into NIR-BODIPY photosensitizers, as versatile and powerful classes of theragnostic agents. They are being explored for expanded applications, including infectious diseases, confirming that NIR-BODIPY derivatives will be foundational to the next generation of precision photomedicine [Contribution 12].

Finally, the work by Villamil et al. examines the direct inhibition of Penicillin-Binding Proteins by boronic acids as a viable complement or alternative to β-lactam therapy. Their perspective supports the potential of boronic acid inhibitors as promising non-β-lactam antimicrobial agents with possible clinical relevance against the loss of β-lactam efficacy and some resistance mechanism of microorganisms involved in human infectious diseases [Contribution 13].

By considering all the reported findings, we agree on the inclusion of B-atoms in new compounds with biological activity and potential action to treat human diseases. In fact, adding B is no longer a question; it is necessary to explore potentially advantageous agents for the prevention, diagnosis, and treatment of human diseases. The emerging pharmacology of BCCs is tangible, although detailed pharmacodynamic, pharmacokinetic, and toxicity data remain limited compared with those for boron-free drug candidates.
